# Preoperative MR Perfusion and Intraoperative MR Imaging in Predicting Surgical Resectability and Quality of Life Outcomes in High-Grade Glioma Patients: A Prospective Cohort Analysis and Case Study

**DOI:** 10.7759/cureus.107306

**Published:** 2026-04-18

**Authors:** Omowumi Oladipo, Clayton Rawson, Kyril L Cole, Julian Brown, Tyler Richards, Karen Salzman, Hunter R Underhill, Michael Karsy, Randy Jensen

**Affiliations:** 1 Neurosurgery, Noorda College of Osteopathic Medicine, Provo, USA; 2 Neurosurgery, School of Medicine, University of Utah, Salt Lake City, USA; 3 Radiology and Imaging Sciences, University of Utah, Salt Lake City, USA; 4 Pediatrics, University of Utah, Salt Lake City, USA; 5 Huntsman Cancer Institute, University of Utah, Salt Lake City, USA; 6 Neurological Surgery, University of Michigan, Ann Arbor, USA; 7 Neurosurgery, University of Utah, Salt Lake City, USA

**Keywords:** glioblastoma, high-grade glioma (hgg), mri, quality of life, surgical planning

## Abstract

Introduction

The successful treatment of high-grade gliomas (HGGs) relies on advanced magnetic resonance (MR) imaging technologies to increase the resection of active tumor areas and impact patient quality of life (QoL) and overall survival (OS). The objective of this prospective pilot study was to evaluate whether preoperative MR perfusion parameters (cerebral blood volume (CBV), cerebral blood flow (CBF)) and intraoperative MRI (iMRI) can predict extent of resection (EOR), OS and progression-free survival (PFS), and postoperative QoL, with a secondary aim of determining whether QoL measures provide prognostic information earlier than conventional neurological or performance status assessments.

Methods

A prospective, single-center study of HGG patients, including grade 3 astrocytoma and glioblastoma, who underwent surgery guided by advanced MRI, was performed. Preoperative CBV, preoperative CBF, and EOR were compared with patient complications and QoL metrics via the MD Anderson Symptom Inventory and Functional Assessment of Cancer Therapy-Brain questionnaires. Patients were divided into good versus poor outcomes by median OS (10 months).

Results

Fourteen patients (mean age: 52.2, 35.7% male), including three isocitrate dehydrogenase 1/2 (IDH1/2) mutant and 11 IDH1/2 wild-type cases, were studied. For patients with good outcomes, OS and PFS were 27 ± 15 months (p = 0.002) and 21 ± 20 months (p = 0.05), respectively. Higher preoperative CBV and CBF were associated with worse OS (p < 0.05). iMRI perfusion was difficult to quantify and limited in correlating with patient outcomes. The mean EOR was 96 ± 5% during iMRIs, and no EOR difference was observed between patients with good or poor survival. Patients with worse OS and PFS showed a significant decline in three-month QoL metrics, especially in social/family well-being domains that preceded changes in Karnofsky Performance Status.

Conclusion

Advanced MRI and quantitative QoL measurements may help predict long-term survival for HGG patients but require further exploration and analysis.

## Introduction

High-grade gliomas (HGG), classified as WHO grades 3 and 4, are largely considered invariably fatal brain tumors. These rare cancers have an estimated incidence of less than 15 cases per 100,000 people annually in the United States (US) [1]. A subset of HGG includes the well-known and aggressive glioblastoma (GBM) type, which is the most common malignant brain tumor in the US. Unsurprisingly, the one- and five-year survival rates for HGG are poor and drop by WHO grade (e.g., five-year survival rates of 40-50% for grade 2, 25-30% for grade 3, and 5-10% for grade 4) [1-3]. Patients with such challenging tumors are often left with significant morbidity and mortality, even with maximal intervention [1-3].

Historically, patient age and the extent of resection (EOR) have been two of the strongest prognostic factors in HGG outcomes. Preoperative Karnofsky Performance Scale scores (KPS) and the degree of necrosis and enhancement on preoperative MRI studies also provide some predictive potential [4]. Increasing EOR is limited by attempts to preserve the functional cortex to prevent worsening patient morbidity. Advanced MR perfusion techniques, such as dynamic susceptibility contrast MRI (DSC-MRI) and dynamic contrast-enhanced MRI (DCE-MRI), have emerged as valuable tools in surgical planning and therapeutic management of various brain tumors, including HGG, by identifying active components of the tumor and postoperative follow-up care [5-7].

The role of MR perfusion in supporting more aggressive EOR remains to be explored. MR perfusion has good sensitivity in some retrospective studies; however, a lack of definitive quantitative thresholds limits application to prospective patient monitoring and long-term prognostication, which may be due to heterogeneity in patient presentation, tumor genotype, and treatment response [8]. The use of patient quality of life (QoL) measures may be an important covariate in assessing the treatment response after MR perfusion-guided resection. The objective of this prospective pilot study was to evaluate whether preoperative MR perfusion parameters (cerebral blood volume (CBV), cerebral blood flow (CBF)) and intraoperative MRI (iMRI) can predict EOR, OS, and PFS. Moreover, postoperative QoL was evaluated with a secondary aim of determining whether QoL measures provide prognostic information earlier than conventional neurological or performance status assessments.

## Materials and methods

Study design and setting

A prospective study of patients receiving treatment for HGG was carried out from 2/15/2017 to 11/29/2019 at the University of Utah, a regional tertiary care center, and the Huntsman Cancer Institute, a National Cancer Institute-designated comprehensive cancer center. A sample size predetermination was not performed as this was a prospective, exploratory study.

Inclusion and exclusion criteria

Included individuals were the following: (1) at least 18 years old, (2) had a preoperative MRI within 14 days of surgical resection, (3) had newly diagnosed T1 contrast-enhancing lesions that were consistent with HGG, (4) had a life expectancy of more than 12 weeks, and (5) were able to provide informed consent. Patients were consecutively screened by the senior author (RLJ) and enrolled at the time the study started (2/15/2017) through the neurosurgery clinic or inpatient service based on the initial MRI scan.

Individuals excluded from the study included those who (1) had received radiation therapy in the past, (2) had another malignancy with a three-year expectation for systemic therapy, (3) required emergent palliative care for a primary disease, (4) showed signs of bleeding diathesis or coagulopathy, (5) had experienced an intracerebral abscess within six months of the surgery date, (6) underwent a major surgical procedure or other open biopsy, (7) suffered a significant traumatic injury within 28 days of the surgery date, (8) were pregnant and/or nursing, or (9) were prisoners.

Imaging protocols and quantitation

Patients underwent preoperative and intraoperative conventional MRI with both DSC perfusion and DCE permeability imaging. MRIs were completed on a 1.5T or 3T Siemens scanner using a phased array, eight-channel head coil. DSC and DCE protocols were optimized according to the manufacturer’s recommendations. DSC was performed during the first pass bolus injection of gadoterate meglumine (0.1 mmol/dg; Gd-Prohance, Guerbet, France) using a power injector (5 mL/s) followed by a 20 mL saline flush.

DSC was acquired using a T2*-weighted single-shot gradient-recalled echo-planar imaging (GRE EPI) sequence with TR/TE, 2070/52 ms; flip angle, 90°; matrix size, 128 × 128; FOV, 220 × 220 mm; slice thickness, 5 mm; 15 axial sections with a 1.5 mm gap; number of excitations (NEX), 1; and acquisition time of 3.5 minutes.

DCE was acquired using a T1 volumetric interpolated examination sequence using the Siemens default tree protocol with slight variations in the protocol based on the machine used. T1 mapping was based on two T1 acquisitions with 2° and 15° flip angles. The most common parameters included TR/TE, 3.07/1.01 ms; flip angle, 20°; matrix size, 96 × 128; FOV 250 × 200 mm; slice thickness, 2 mm; 32 axial sections with a 0 mm gap; number of excitations (NEX), 1; and acquisition time of 7 min 23 s. Contrast injection of gadobutrol (0.1-mmol/kg bolus; Gadovist, Bayer Schering Pharma, Berlin, Germany) was followed by 3 mL/s saline flush.

Data were reconstructed using Olea Sphere 3.0 (Olea Medical, La Ciotat, France). For DSC, analysis was performed using the circular singular value decomposition algorithm for deconvolution. The arterial input function was selected automatically using the software’s cluster analysis algorithm. Motion correction and smoothing were applied for all subjects. For DCE analysis, modeling was based on the extended Tofts model [9]. Imaging analysis was performed by a single technician who was unaware of the patient’s pathology at the time measurements were performed.

For quantitative imaging, T1 post-contrast imaging was co-registered with the DSC and DCE images, where a circular region of interest (ROI) (diameter 7 mm) was drawn on four nonoverlapping areas of the apparent highest plasma volume (Vp) within the tumor on the DCE post-processed images. ROIs were projected to the same location on the co-registered DSC perfusion maps. The parameters Vp, Ktrans, and area under the curve (AUC) were recorded for DCE, where leakage-corrected relative cerebral blood volume (rCBV) and relative cerebral blood flow (rCBF) were recorded for DSC. Enhancing tumor, surrounding fluid-attenuated inversion recovery (FLAIR) hyperintense signal abnormality, and apparent diffusion coefficient (ADC) were also measured.

Preoperative assessment

MR perfusion imaging and preoperative MRI were performed on the patients studied. Using Olea Medical software, rCBV and rCBF were calculated. QoL measurements, postoperative neurological evaluation, and adverse event assessments were also collected. QoL was measured through the MD Anderson Symptom Inventory (MDASI) (Table 1) and the Functional Assessment of Cancer Therapy-Brain (FACT-Br) (Table 2) questionnaires [10,11]. Standard neurological assessments were performed preoperatively and during follow-up. Common Terminology Criteria for Adverse Events (CTCAE) were all evaluated. A CTCAE ≥ 3 grade event was considered a severe adverse event. Neurological exams were performed preoperatively, postoperatively, and at one-, three-, six-, and 12-month follow-up. QoL measurements were performed at all time points except for immediately postoperatively. Assessments were generally performed at clinical follow-up time points, and for incomplete data, a clinical coordinator contacted the patient at least three times to attempt completion of the required metric. Surveys were delivered to patients in paper form, filled out at clinic visits. The results were not reviewed by the surgeon, and the results were subsequently entered into a database by a study coordinator.

**Table 1:** MDASI-Br Quality of Life Metric Recreated with permission from Armstrong et al. [10]. Severity of symptoms based on a scale of 0-10 (0 = not present through 10 = as bad as you can imagine). Patients select the appropriate number for each item. Abbreviation: MDASI, MD Anderson Symptom Inventory

MDASI-Br quality of life metric
1. Your pain at its worst?
2. Your fatigue (tiredness) at its worst?
3. Your nausea at its worst?
4. Your disturbed sleep at its worst?
5. Your feeling of being distressed (upset) at its worst?
6. Your shortness of breath at its worst?
7. Your problem with remembering things at its worst?
8. Your problem with lack of appetite at its worst?
9. Your feeling drowsy (sleepy) at its worst?
10. Your having a dry mouth at its worst?
11. Your feeling sad at its worst?
12. Your vomiting at its worst?
13. Your numbness or tingling at its worst?
14. Your weakness on one side of the body at its worst?
15. Your difficulty understanding at its worst?
16. Your difficulty speaking (finding the words at its worst?

**Table 2:** FACT-Br Quality of Life Metric Recreated with permission from Lien et al. [11]. Patients rate each statement using a scale of 0-4, where 0 is not at all and 4 is very much considering their experience of each symptom in the past seven days. Abbreviation: FACT-BR, Functional Assessment of Cancer Therapy-Brain

Physical well-being
I have a lack of energy.
I have nausea.
Because of my physical condition, I have trouble meeting the needs of my family.
I have pain.
I am bothered by side effects of treatment.
I feel ill.
I am forced to spend time in bed.
	Emotional well-being
I feel sad.
I am satisfied with how I am coping with my illness.
I am losing hope in the fight against my illness.
I feel nervous.
I worry about dying.
I worry that my condition will get worse.
Social/family well-being
I feel close to my friends.
I get emotional support from my family.
I get support from my friends.
My family has accepted my illness.
I am satisfied with family communication about my illness.
I feel close to my partner.
I am satisfied with my sex life.
Functional well-being
I am able to work (including work at home).
My work (including work at home) is fulfilling.
I am able to enjoy life.
I have accepted my illness.
I am sleeping well.
I am enjoying the things I usually do for fun.
I am content with the quality of my life right now.

Inoperative assessment

Maximal resection of all T1 with contrast-enhancing volume, defined as the active tumor, was planned for each case. iMRI was obtained after the primary surgeon determined that a maximal safe resection was performed. The surgeon’s estimate of EOR was acquired prior to the iMRI scan. Neuronavigation based on preoperative and intraoperative imaging was performed (Medtronic S7) to guide surgical resection. Additional resection of the T1 contrast-enhancing tumor was performed if necessary based on iMRI data, and the final EOR was assessed by comparing preoperative and postoperative imaging. A neuropathologist examined all frozen, permanent, and research pathology samples, and a patient’s diagnosis of HGG was confirmed using the WHO 2021 criteria.

Postoperative assessment

The ellipsoid method (a × b × c × 1/3) was used to measure intraoperative and postoperative T1-weighted contrast-enhanced and T2/FLAIR tumor volumes. EOR was then analyzed using the formula: (preoperative volume - postoperative volume) / preoperative volume × 100. Tumor location, laterality, and infiltration were evaluated.

Statistical analysis

To evaluate continuous variables, a t-test was used; to evaluate discrete variables, a chi-square test was performed. Means and standard deviations (SD) were calculated for continuous variables. Using SPSS (IBM Corp., Armonk, NY) and RStudio (Posit, Boston, MA), statistical analyses were carried out, with p < 0.05 being considered significant. Imputation was performed for quantitative perfusion variables using the group median value. 

Overall survival (OS) and progression-free survival (PFS), which were censored if any patient reached five years of follow-up, were assessed using Kaplan-Meier survival curves. The Cox proportional hazards model was employed for the multivariable analysis of OS and PFS. Regarding OS and PFS prediction, the effectiveness of MR perfusion variables was assessed using receiver operating characteristic (ROC) curves. Patients were divided into good and poor outcomes by use of the median OS (defined as >10 months of survival).

## Results

Demographics

A total of 14 eligible patients (mean age 52.2 years, 35.7% male) were included in the final analysis (Table 3). Patients were divided by median OS for the group (10 months) into patients with poor outcomes (OS: 4 ± 4 months) vs. good outcomes (27 ± 15 months) (p = 0.002). There were no significant differences in age (p = 0.3) or sex (p = 0.6). No significant differences were found between IDH1/2 wildtype and IDH1/2 mutant status (p = 0.5) or WHO grade (p =0.5). No differences in other biomarkers such as p53 (p = 0.2), PTEN (p = 0.3), and MGMT statuses (p = 1.0) were seen. Tumors in the frontal lobe but no other locations were more commonly associated with poor outcomes (p = 0.05). No difference in hospital floor (p = 0.4) and intensive care unit (p = 0.6) length of stay (LOS) was seen between poor and good outcome groups.

**Table 3:** Patients’ Demographics and Baseline Characteristics Abbreviations: IDH: isocitrate dehydrogenase; LOS: length of stay; MGMT: O6-methylguanine-DNA methyltransferase; P53: tumor protein 53; PTEN: phosphatase and tensin homolog; WHO: World Health Organization

Characteristics	Poor outcome (n = 7)	Good outcome (n = 7)	p-value
Age, years	48.29 ± 17.75	57.19 ± 17.08	0.3
Sex, male	4 (57.1%)	5 (71.4%)	0.6
Tumor			
WHO grade 3	2 (40.0%)	1 (14.2%)	0.5
WHO grade 4	5 (60.0%)	6 (85.7%)	
Mutation type			
IDH1/2 wildtype	6 (85.7%)	5 (71.4%)	0.5
IDH1/2 mutant	1 (14.3%)	2 (28.6%)	
P53	4 (57.1%)	6 (85.7%)	0.2
PTEN	0 (0.0%)	1 (14.2%)	0.3
MGMT	3 (42.9%)	3 (42.9%)	1.0
Tumor location			
Frontal	3 (42.9%)	0 (0.0%)	0.05
Parietal	1 (28.6%)	2 (28.6%)	1.0
Temporal	3 (42.9%)	3 (42.9%)	1.0
Occipital	0 (0.0%)	2 (28.6%)	0.1
Hospital LOS			
Hospital floor	4.5 ± 7.6	1.8 ± 1.0	0.40
Intensive care unit	3.3 ± 0.5	3.6 ± 1.0	0.60

Survival analysis of MR perfusion parameters

An OS of 4 ± 4 vs. 27 ± 15 months (p = 0.002) and PFS of 4 ± 4 vs. 21 ± 20 months (p = 0.05) were seen for patients with poor vs. good outcomes, respectively (Table 4). Postoperative complications, including neurological injury (p = 0.3) and deep vein thrombosis (DVT) (p = 0.3), were not significantly different between groups. Neurological deficits were not significantly different between groups preoperatively (p = 0.5), at one-month postoperative (p = 0.3), and at three-month postoperative (p = 0.2). Comparison of the neurological exam was not possible at six- and 12-month postoperative due to the limited number of patients in the poor outcome group.

**Table 4:** Perioperative Outcomes Following MR-Perfusion Evaluated Tumors Abbreviations: DVT, deep vein thrombosis; PFS, progression-free survival; OS, overall survival

Characteristics	Poor outcome (n = 7)	Good outcome (n = 7)	p-value
OS, months	4 ± 4	27 ± 15	0.002
PFS, months	4 ± 4	21 ± 20	0.05
Intraoperative neurological injury	1 (14.3%)	0 (0.0%)	0.3
DVT	1 (14.3%)	0 (0.0%)	0.3
Neurological exam deficits			
Preoperative	2 (28.6%)	1 (14.3%)	0.5
One-month postoperative	2 (66.7%)	2 (28.6%)	0.3
Three-months postoperative	2 (100.0%)	3 (50.0%)	0.2
Six-months postoperative	0 (0.0%)	0 (0.0%0	-
12-months postoperative	0 (0.0%)	2 (40.0%)	-

QoL evaluation

**Figure 1:**
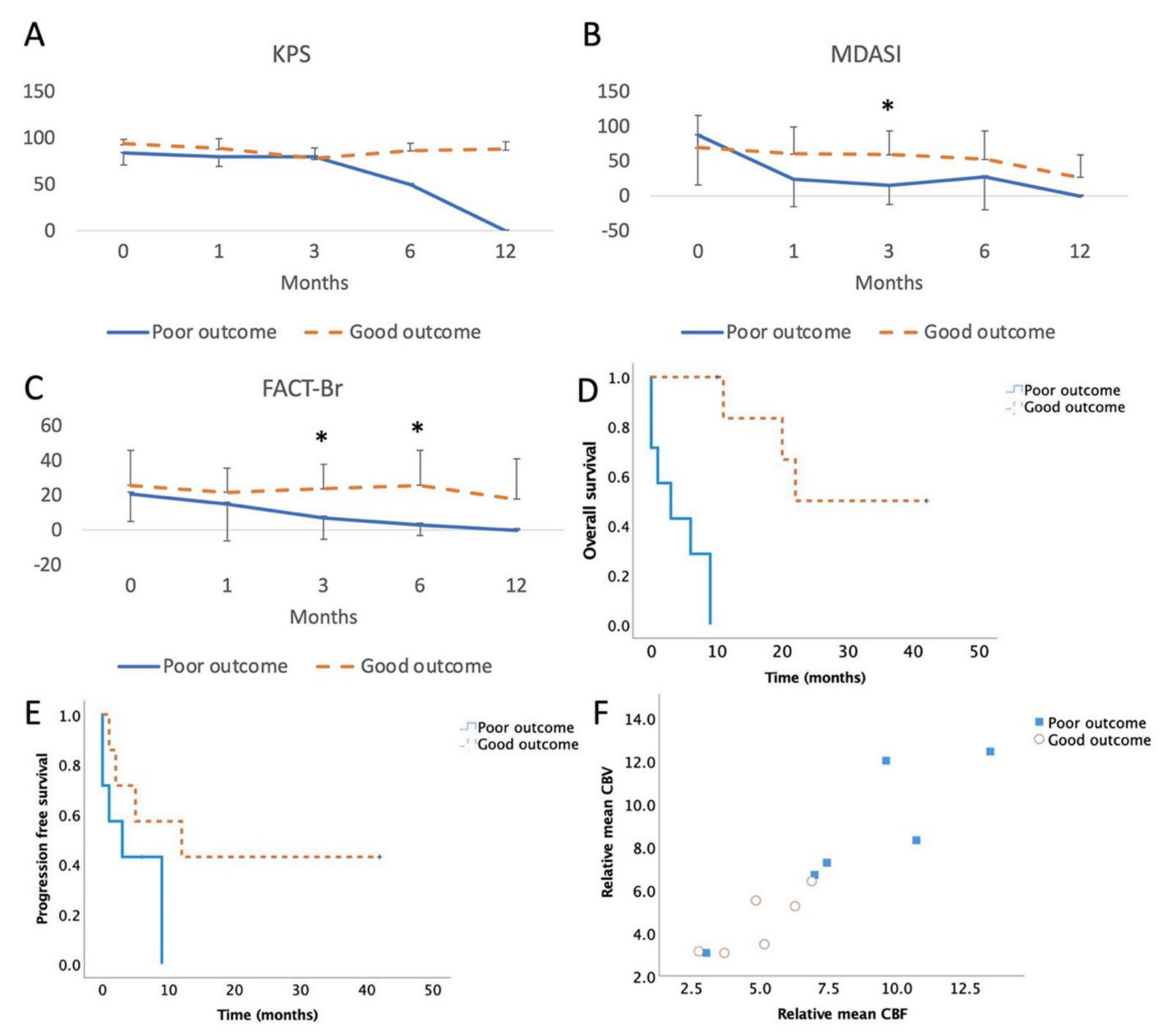
Evaluation of Survival and QoL Changes in Glioma Patients Undergoing Resection With iMRI Comparison of QoL measures between patients with poor vs. good outcomes, as separated by median overall survival, was evaluated. (A No significant difference in KPS was seen between groups at any time point. (B) MDASI scores were significantly worse for poor outcome patients at three months (p = 0.02). (C) FACT-Br scores were lower for worse outcome patients at three months (p = 0.03) and six months (p = 0.01). (D) OS was significantly worse in poor outcome patients (log-rank test, p = 0.0001). (E) PFS did not show any significant difference between patient groups. (F) Correlation of relative mean CBV and CBF demonstrates lower numbers for patients with poor outcomes. Abbreviations: FACT-BR: Functional Assessment of Cancer Therapy-Brain questionnaire; iMRI, intraoperative MRI; MDASI: MD Anderson Symptom Inventory; QoL, quality of life

Postoperative QoL metrics showed an initial decline, with recovery generally observed by three months post-surgery. Patients with limited improvement in QoL had significantly worsened OS and PFS, with a decline in QoL preceding a decline in KPS (Figure 1). Overall QoL measures showed a gradual decline for KPS, MDASI, and FACT-Br measures (Table 5). Differences in KPS scores for patients with poor outcomes were first identified at six months (Figure 1A), while those for MDASI (Figure 1B) and FACT-Br (Figure 1C) were seen at three months. MDASI scores showed a significant decrement at three months in patients with poor outcomes (p = 0.02), while FACT-Br scores were worse at three and six months in patients with poor outcomes (p = 0.03, p = 0.01). Expectedly, Kaplan-Meier curves of OS (log-rank, p = 0.0001, Figure 1D) but not PFS (log-rank, p = 0.1, Figure 1E) were significantly worse for poor survival.

**Table 5:** QoL Measures After Exclusion of Patients With Major Complications Abbreviations: FACT-BR: Functional Assessment of Cancer Therapy-Brain questionnaire; KPS: Karnofsky Performance Scale; MDASI: MD Anderson Symptom Inventory

KPS, mean ± SD	
Preoperative KPS	94 ± 5
One-month KPS	89 ± 11
Three-month KPS	78 ± 12
Six-month KPS	87 ± 8
12-month KPS	88 ± 8
MDASI, mean ± SD	
Preoperative MDASI	70 ± 46
One-month MDASI	61 ± 39
Three-month MDASI	60 ± 34
Six-month MDASI	53 ± 41
12-month MDASI	27 ± 33
FACT-BR, mean ± SD	
Preoperative FACT-BR	26 ± 20
One-month FACT-BR	22 ± 14
Three-month FACT-BR	24 ± 14
Six-month FACT-BR	26 ± 20
12-month FACT-BR	18 ± 23

**Figure 2:**
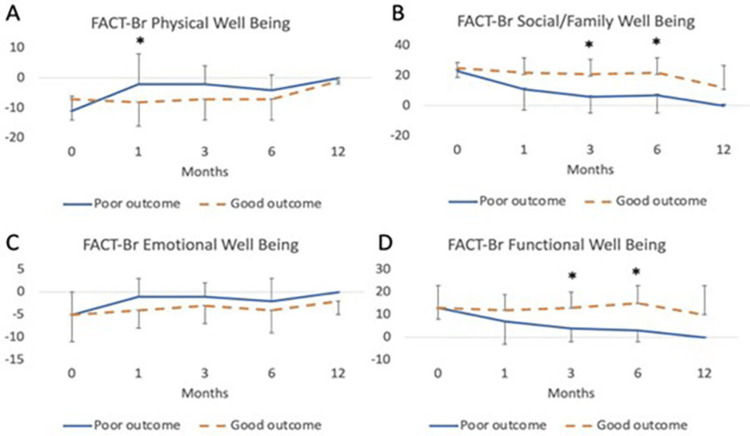
Evaluation of FACT-BR Subdomain QoL Measures in Glioma Patients Undergoing Resection With iMRI Subdomain scores of the FACT-Br were compared in patients with good and poor outcomes. (A) Physical well-being scores were divided between patient groups at one month (p = 0.02). (B) Social and family well-being scores differed between groups at three months (p = 0.02) and six months (p = 0.03). (C) Emotional well-being did not show any difference between groups at various time points. (D) Functional well-being differed between groups at three months (p = 0.03) and six months (p = 0.005). Abbreviations: FACT-BR: Functional Assessment of Cancer Therapy-Brain questionnaire; iMRI, intraoperative MRI; QoL, quality of life

Subdomains of FACT-Br were evaluated, showing significant differences in physical well-being at 1 month (Figure 2A, p = 0.02), and social/family well-being at three and six months (Figure 2B, p = 0.02 and p = 0.03, respectively). No difference in emotional well-being was seen between outcome groups (Figure 2C). Function well-being showed worsening scores at three and six months between groups (Figure 2D, p = 0.03 and p = 0.005, respectively).

Preoperative and tumor volume measurements

OR time was significantly longer in the good outcome group (442.8 ± 21.4 minutes) compared to the poor outcome group (406.7 ± 33.4 minutes) (p = 0.03) (Table 6). Estimated blood loss was no different between poor and good outcome groups (p = 0.5). The mean overall EOR observed during iMRIs was 96 ± 5%. Estimated pre-iMRI resection (p = 0.4), T1 enhancing EOR (p = 0.06), and hyperintense FLAIR tumor volume (p = 0.4) did not show a difference between poor and favorable outcomes.

**Table 6:** Perioperative and MR Perfusion Measurements Comparison of perioperative and MR perfusion measurements between cohort groups (i.e., poor outcome and good outcome). Abbreviations: ADC: apparent diffusion coefficient; AUC: area under the curve; CBF: cerebral blood flow; CBV: cerebral blood volume; EOR: extent of resection; FLAIR: fluid-attenuated inversion recovery; iMRI: intraoperative MRI; K2: second-order rate constant; Kep: rate constant (backflux from Ve to Vp); Ktrans: volume transfer constant; OR: operating room; Ve: extravascular extracellular volume fraction; Vp: plasma volume fraction

Characteristic	Poor outcome (n = 7)	Good outcome (n = 7)	p-value
OR time (minutes)	406.7 ± 33.4	442.8 ± 21.4	0.03
Estimated blood loss (mL)	214 ± 75	257 ± 127	0.5
Percent resection estimated before iMRIs	86% ± 29%	96% ± 5%	0.4
Enhancing T1 tumor volume (cm^3^)			
Preoperative	15.95 ± 11.99	20.16 ± 17.33	0.6
Postoperative	1.27 ± 1.61	0.24 ± .30	0.2
EOR	89.5 ± 10.8	98.7 ± 1.5	0.06
Hyperintense FLAIR tumor volume (cm^3^)			
Preoperative	87.20 ± 60.09	102.34 ± 69.53	0.7
Postoperative	54.13 ± 62.85	42.69 ± 39.49	0.8
EOR	28.6 ± 54.1	51.5 ± 28.9	0.4
Perfusion measurements			
CBV	8.30 ± 3.52	4.50 ± 1.42	0.05
CBF	8.54 ± 3.56	4.95 ± 1.54	0.03
K2	-716.21 ± 516.38	-290.72 ± 577.36	0.2
Vp	247.76 ± 100.75	241.88±198.74	0.9
Ve	716.20 ± 208.18	666.80 ± 322.02	0.8
AUC	30328.51 ± 13232.09	24467.39 ± 12568.40	0.4
K trans	267.31 ± 219.47	1427.94 ± 12568.40	0.3
Kep	4 (57.1%)	6 (85.7%)	0.2
ADC	844.54 ± 245.12	734.05 ± 450.67	0.8

**Figure 3:**
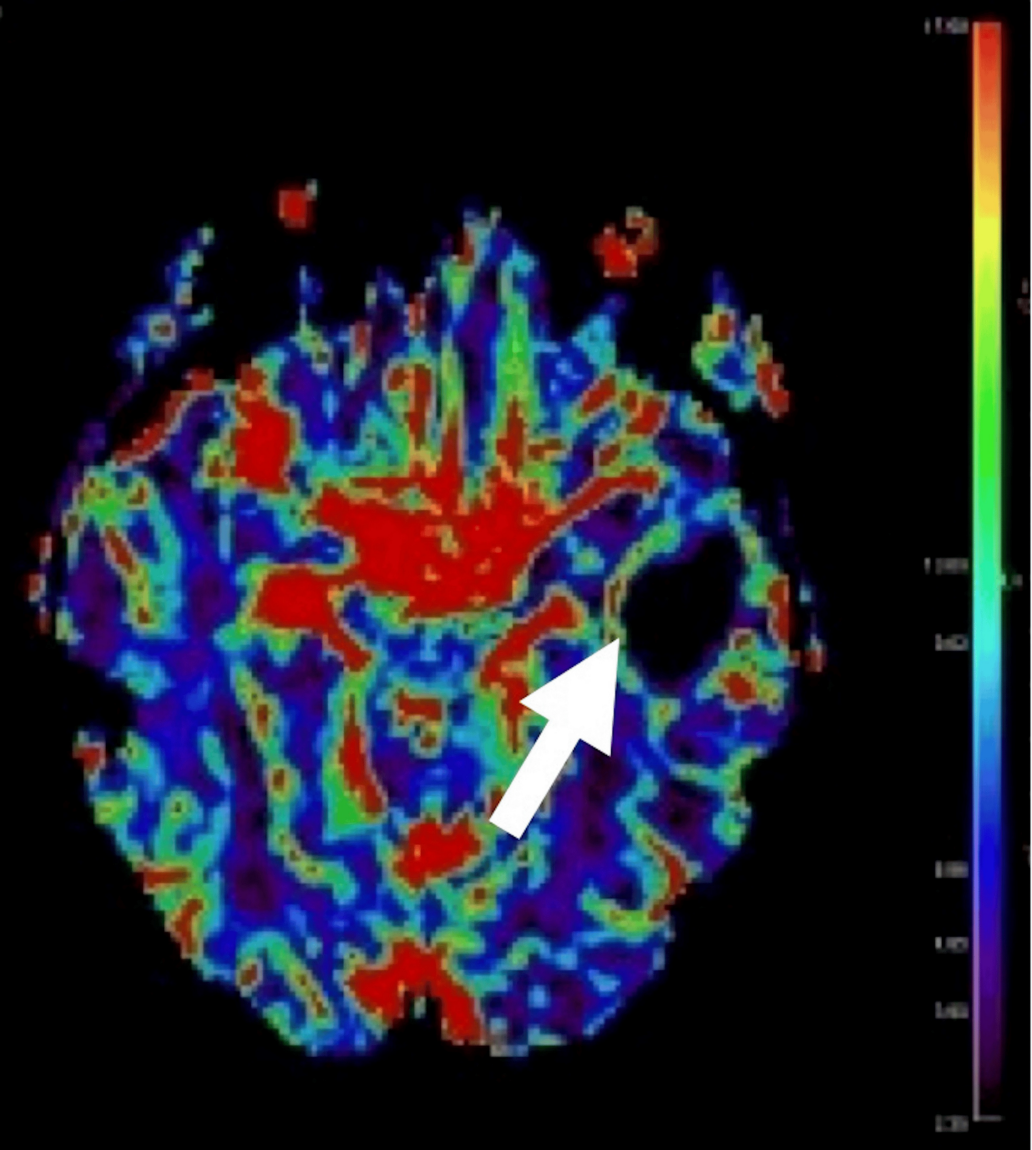
Preoperative Cerebral Blood Flow Analysis of Left Temporal Tumor Increased cerebral blood flow surrounding a left temporal necrotic high-grade glioma was seen (arrow).

**Figure 4:**
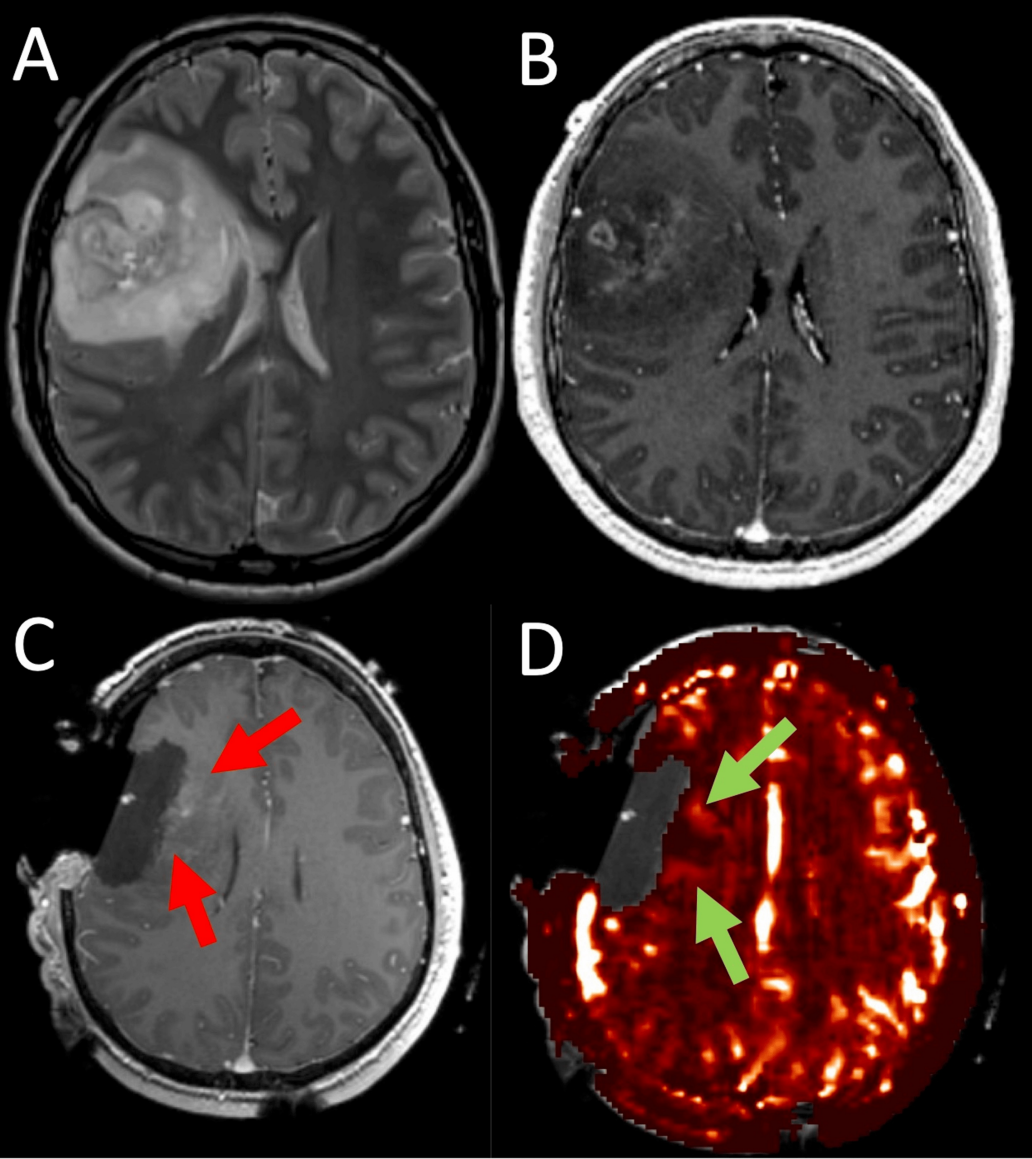
Preoperative MRI, Intraoperative MRI, and Intraoperative Perfusion of Anaplastic Oligodendroglioma A) Preoperative axial T2 and B) T1 contrast-enhanced imaging demonstrate a right frontal anaplastic oligodendroglioma with mass effect and some patchy enhancement. C) Intraoperative T1 contrast-enhanced imaging shows a resection cavity with some faint perilesional enhancement (red arrows). D) Axial dynamic contrast-enhanced perfusion with quantification of Vp overlayed on a T1 contrast-enhanced image shows some faint increased plasma volume (green arrows).

Preoperative perfusion analysis revealed a significantly lower CBF in the good outcome group (4.95 ± 1.54) compared to the poor outcome group (8.54 ± 3.56, p = 0.03) (Figure 1E). Similarly, lower CBV in the good outcome group (4.5 ± 1.42) compared with poor outcomes (8.3 ± 3.52, p = 0.05). No significant quantitative differences were observed in other preoperative or iMRI perfusion parameters, including K2, Vp, Ve, AUC, K trans, Kep, and ADC (Table 6). Preoperative MRI perfusion analysis yielded significant benefit for CBF and CBV, but was more apparent on quantitative rather than qualitative analysis (Figure 3). iMRI perfusion analysis yielded limited results (Figure 4).

## Discussion

Our results showed an association of higher CBF and CBV with relatively worse outcomes in HGG. High rates of EOR were expectedly seen in this modern series of HGG, limiting the utility of intraoperative MR perfusion. Moreover, worsened QoL measures between one and six months were associated with worse outcomes and more sensitive than KPS in predicting outcome. Subdomains of FACT-Br demonstrated physical well-being scores at one month, and social/family as well as functional well-being scores at three and six months were associated with worse outcomes. Reduction in QoL measurements was identified in patients with worsened outcomes and could be identified at three-month postoperative visits, well before neurological deficits or decline in KPS were identified. Moreover, worsened QoL was identified regardless of other changes in imaging, clinical, or biochemical factors. These results demonstrate the association of various clinical, radiographic, and QoL metrics in impacting patient survival.

Several studies have evaluated MR perfusion as a method to differentiate HGG from radionecrosis [12,13] or distinguish benign from malignant lesions [14]. While CBV and CBF have supportive evidence for predicting prognosis and clinical progression of HGG, integration of MR perfusion for routine clinical use remains controversial [8]. Limited sensitivity and specificity of MR perfusion, combined with its reliance on clinical interpretation, can impact its usefulness. Our results showed that preoperative CBF and CBV were greater in patients who failed to achieve good outcomes following HGG resection, defined as survival greater than 10 months (p < 0.05). These results are similar to prior studies [8,15].

Integration of MR perfusion with other clinical parameters may improve survival prediction. Patel et al.’s [8] study of 45 patients with GBM who underwent MR perfusion showed worsened OS and PFS with increasing CBV of non-enhancing tumor areas. The authors demonstrated that KPS, EOR, and epidermal growth factor receptor mutation impacted OS. The combined EOR with marginal CBV did not impact prognosis, but inclusion of CBV, age, and KPS could help with survival prediction. CBV may play a role in outcome prediction, but it may require additional parameters to be clinically useful.

Our results similarly showed a limited impact for other perfusion parameters other than CBF and CBV. In addition, a limited role for clinical or mutational factors on prognosis was seen. Both groups achieved high EORs with no differences in the rate of operative complications and were expectedly IDH1/2 wildtype. These results suggest the safety of iMRI and MR perfusion despite limited clinical utility for all parameters at this stage.

QoL measurements demonstrated potential declines at one to three months that preceded neurological exam changes or changes in KPS. Domains related to social/family well-being and functional well-being were the most impactful on the outcome. QoL subdomain changes in physical well-being were only seen at one month, while those for social/family and functional well-being were seen at three and six months. Due to the limited sample size of this pilot study and patient mortality at later time points, additional differences beyond six months were not statistically significant. Our results are comparable to prior studies, indicating the reliability of these QoL measures on predicting disease severity, symptom progression, and OS in primary brain tumors [10,11,16,17]. Moreover, the impact of specific subdomains, such as spiritual well-being, has been reported in glioma [18]. These data indicate that the assessment of QoL in HGG patients may have a role in predicting patient outcomes in both preoperative and postoperative settings. As many patients with HGG often experience cognitive decline and functional impairment, the use of QoL measures may help providers understand and integrate how various treatments affect the emotional and social well-being of their patients beyond survival rates [19]. Using QoL metrics in routine practice may offer insights that can better guide therapeutic decision-making. The differences in QoL measures may also be important to consider when evaluating prognosis in clinical, radiological, and genomic/biomarker studies. The use of QoL measurements in the clinical management of HGG requires further work, but may be a potent way of evaluating other biomarkers and treatment modalities.

This exploratory study was approved with a small sample size, aiming to evaluate feasibility and safety rather than being powered for a clinical endpoint. Further assessment in larger cohorts of QoL measures may be useful in defining their role in predicting outcomes. Greater frequency of sampling may also be useful in assessing whether changes in QoL over time correlate with outcomes. The sensitivity/specificity of MR perfusion data may also be improved through the integration of machine learning for the analysis of perfusion maps. Integrating further genetic biomarkers and imaging parameters with QoL measures could help predict outcomes.

Limitations

Despite the prospective nature of this study, some limitations were identified. Notably, MR perfusion quantitation required significant post-processing of data and could not be used to identify tumor hotspots or a priori select areas of interest in real time. Only CBF/CBV heatmaps were available for interpretation in guiding treatment preoperatively. iMRI perfusion quantitation remained limited for clinical use or correlation to outcome. The imaging and surgical management, despite being delineated prior to the study initiation, were still challenging to implement given the heterogeneous presentations, imaging features, and management strategies of HGG. The relatively small sample size may also limit the generalizability of these findings and the ability to study subgroups of patients. Missing data were seen in a very small subset of continuous variables, and robust methods to track follow-up QoL data were used. However, the small sample size of this study may be impacted by missing data.

## Conclusions

This study sought to evaluate MR perfusion parameters and QoL measures as predictors of outcomes following resection of HGG. CBF and CBV showed an association with outcomes. However, postoperative changes in FACT-BR and MDASI scores were also acutely sensitive to worsened prognosis. The combined integration of clinical, imaging, and QoL measures will be necessary to study patient prognosis in HGG.
